# Contribution of *BDNF* and *DRD2* genetic polymorphisms to continued opioid use in patients receiving methadone treatment for opioid use disorder: an observational study

**DOI:** 10.1186/s13722-015-0040-7

**Published:** 2015-10-06

**Authors:** Monica Bawor, Brittany B. Dennis, Charlie Tan, Guillaume Pare, Michael Varenbut, Jeff Daiter, Carolyn Plater, Andrew Worster, David C. Marsh, Meir Steiner, Rebecca Anglin, Dipika Desai, Lehana Thabane, Zainab Samaan

**Affiliations:** MiNDS Neuroscience Program, McMaster University, Hamilton, ON Canada; Population Genomics Program, Chanchlani Research Centre, McMaster University, Hamilton, ON Canada; Peter Boris Centre for Addictions Research, St. Joseph’s Healthcare Hamilton, Hamilton, ON Canada; Department of Clinical Epidemiology and Biostatistics, McMaster University, Hamilton, ON Canada; Michael G. DeGroote School of Medicine, McMaster University, Hamilton, ON Canada; Department of Pathology and Molecular Medicine, McMaster University, Hamilton, ON Canada; Canadian Addiction Treatment Centres (CATC), Richmond Hill, ON Canada; Department of Medicine, McMaster University, Hamilton, ON Canada; Northern Ontario School of Medicine, Laurentian Campus, Sudbury, ON Canada; Department of Psychiatry and Behavioural Neurosciences, McMaster University, Hamilton, ON Canada; Women’s Health Concerns Clinic, St. Joseph’s Healthcare Hamilton, Hamilton, ON Canada; Department of Obstetrics and Gynecology, McMaster University, Hamilton, ON Canada; Biostatistics Unit, Centre for Evaluation of Medicine, Hamilton, ON Canada; System Linked Research Unit, Hamilton, ON Canada; Mood Disorders Program, St. Joseph’s Healthcare Hamilton, 100 West 5th St., Hamilton, ON L8N 3K7 Canada

**Keywords:** Opioid use disorder, Methadone maintenance treatment, Treatment response, BDNF, Val66Met, DRD2

## Abstract

**Background:**

The heritability of opioid use disorder has been widely investigated; however, the influence of specific genes on methadone treatment outcomes is not well understood. The association between response to methadone treatment and genes that are involved in substance use behaviors and reward mechanisms is poorly understood, despite evidence suggesting their contribution to opioid use disorder. The aim of this study was to investigate the effect of brain-derived neurotrophic factor (*BDNF*) and dopamine receptor D2 (*DRD2*) polymorphisms on continued opioid use among patients on methadone treatment for opioid use disorder.

**Methods:**

*BDNF* 196G>A (*rs6265*) and *DRD2*-241A>G (*rs1799978*) genetic variants were examined in patients with opioid use disorder who were recruited from methadone treatment clinics across Southern Ontario, Canada. We collected demographic information, substance use history, blood for genetic analysis, and urine to measure opioid use. We used regression analysis to examine the association between continued opioid use and genetic variants, adjusting for age, sex, ethnicity, methadone dose, duration in treatment, and number of urine screens.

**Results:**

Among 240 patients treated with methadone for opioid use disorder, 36.3 percent (n = 87) and 11.3 percent (n = 27) had at least one risk allele for *rs6265* and *rs1799978*, respectively. These genetic variants were not significantly associated with continued opioid use while on methadone maintenance treatment [*rs6265*: odds ratio (OR) = 1.37, 95 % confidence interval (CI) = 0.792, 2.371, *p* = 0.264; *rs1799978*: OR 1.27, 95 % CI 0.511, 3.182, *p* = 0.603].

**Conclusions:**

Despite an association of *BDNFrs6265* and *DRD2rs1799978* with addictive behaviors, these variants were not associated with continued illicit opioid use in patients treated with methadone. Problematic use of opioids throughout treatment with methadone may be attributed to nongenetic factors or a polygenic effect requiring further exploration. Additional research should focus on investigating these findings in larger samples and different populations.

## Background

Rates of illicit opioid use are continuing to rise on a global scale, with North America being among the regions with most problematic levels of opioid use [1, 2]. Now identified as a growing public health problem, the use of illicit opioids is putting individuals at risk for opioid-related problems, including psychological and physical dependence. The development of opioid use disorder is influenced by a combination of environmental, behavioral, and biological factors, which contribute to the chronic and relapsing nature of the illness. Treatment for opioid use disorder with methadone, a synthetic opioid agonist, has been shown to be effective in reducing rates of relapse [3, 4]; however, there are a number of patients who continue to abuse opioids while in treatment, with little to no progress in their recovery.

The success of opioid agonist treatments is likely to be influenced by individual differences in gene profiles [5]. Evidence for the heritability of opioid use disorders has long been established [6–14], from which an interest in the specific genetic variability of opioid use disorder and methadone maintenance treatment (MMT) has evolved [5, 15]. Existing genetic studies have explored the therapeutic response to MMT, with a focus on opioid use relapse and methadone dosing. Opioid receptor genes, specifically *OPRM1*, and methadone metabolism genes, including *ABCB1* and *CYP450,* are among the most commonly studied genes to date [16–22]. However, the association between methadone treatment response and other genes such as those involved in substance use behaviors and reward mechanisms remains unknown, despite evidence suggesting their contribution to opioid use disorder [23, 24].

The brain-derived neurotrophic factor (*BDNF*) gene encodes the neurotrophic protein, BDNF, which modulates neuron survival and neurotransmission [25]. Located on chromosome 11p13-15, *BDNF* has been identified as a strong candidate gene in multiple psychiatric and substance use disorders [26–29], including opioid use disorder [30–32], as well as for certain addictive behaviors such as drug seeking, impulsivity, polysubstance use, and cigarette smoking [33–35]. The *BDNF* 196G>A single nucleotide polymorphism (SNP) *rs6265*, also known as Val66Met, is found in the pro-*BDNF* region of the gene and inhibits secretion of the BDNF protein. Val66Met has been linked with deficits in neurotrophin and neurotransmitter release in specific areas that are responsible for behavior, learning, and memory [36, 37]. In the context of methadone treatment, *BDNF* has been explored in relation to BDNF plasma levels [30] and methadone dose [38], with only one study examining methadone treatment response to date [39]. In their study of 91 patients enrolled in an MMT program, de Cid and colleagues found that a haplotype block in the *BDNF* genomic region (GenBank accession number NC_000011; including 21 polymorphisms in a 63.8 kb region of coding sequence, and 3′ and 5′ untranslated regions) containing this specific SNP was more frequent in nonresponders compared to responders. However, the generalizability of these findings is limited by small sample size, large confidence intervals (CIs), and short period of urinalysis testing (previous four urine screens) [39].

Similarly, the dopamine receptor D2 (*DRD2*) gene plays a major role in opioid use disorders because of its involvement in the reward–dependence pathway [40]. The *DRD2* gene is localized to chromosome 11q23 and is responsible for the synthesis of dopamine D2 receptors, which are involved in many signaling and neurotransmission processes underlying addiction, including motivation, pleasure, and reward. A reduction in dopamine receptor signaling has been linked to reward deficiency syndrome, whereby continuous use of opioids acts to compensate for this inhibited dopamine release or “low reward” state [41]. The dopaminergic system mediates withdrawal and drug-related learning [42] and is therefore an important candidate gene for studying opioid use and methadone treatment response. To date, most of the addiction literature involving *DRD2* has focused on the *Taq1A* (*rs1800497*) polymorphism [43–46]. There is also widespread evidence for an effect of *Taq1A* on methadone dose, metabolism, and response, which is most often associated with poor outcomes [24, 40, 43, 47, 48]. However, as the *DRD2* gene is heavily involved in the activation of dopamine reward circuitry, it is likely that other SNPs that have not been investigated as extensively as *Taq1A* are associated with methadone treatment outcomes. A promising target polymorphism, *DRD2*-241A>G (*rs1799978*), is of particular interest, as it has shown preliminary evidence for an association with opioid use disorder and methadone dose in a sample of 85 German drug users admitted to an outpatient methadone treatment center [43].

Despite evidence for a strong association with addictive and reward behaviors, few studies of *BDNF rs6265* and *DRD2 rs1799978* in the context of opioid dependence and response to methadone treatment are available, and those are often limited by small samples or variation in the definitions of methadone treatment response. Based on existing literature, there is high potential for these SNPs to demonstrate an effect on methadone treatment response, which may have important implications for treatment prognosis. The current study aims to examine the genetic contribution to methadone treatment response (continued opioid use) in individuals with opioid use disorder, with a specific focus on addiction-related genes, *BDNF* and *DRD2*. We hypothesize that carriers of the minor alleles of both *rs6265* and *rs1799978* will be more likely to engage in continued illicit opioid use during methadone treatment, indicating poor treatment response.

## Methods

We have reported detailed methods of this study sample previously [49]. Data used in this study were collected as part of the GENetics of Opioid Addiction (GENOA) research program, in collaboration with Canadian Addiction Treatment Centres (CATC; formerly known as Ontario Addiction Treatment Centres, or OATC) and the Population Genomics Program at McMaster University. This study is a cross-sectional analysis of men and women with a DSM-IV opioid dependence disorder, recruited consecutively from four outpatient methadone clinics across Southern Ontario between June and December of 2011. This study was approved by the Hamilton Integrated Research Ethics Board (HIREB), and written informed consent was obtained from each participant.

Participants were included in the study if they were ≥18 years of age, enrolled in a methadone treatment program at the CATC clinics, on a stabilized dose for the past 3 months, and able to provide consent and blood samples. We utilized the genetic information from 240 participant blood samples from the GENOA study in this investigation, in addition to substance use and medical history obtained through structured clinical interviews.

Illicit opioid use (referring to the use of illegal opioids, such as heroin, or using prescription painkillers that were not prescribed for the given individual/condition) was detected by regular urine screens (weekly/biweekly) and measured as the percentage of positive urine screens per total number of urine screens available. Participants with <80 % negative opioid urine screens were classified as using illicit opioids during treatment, or as treatment nonresponders. We also collected information on demographics, methadone treatment duration, methadone dose, age of initial opioid use, and psychiatric history.

### SNP selection and genotyping

We selected the *BDNF* and *DRD2* genes on the basis of evidence supporting their involvement in opioid dependence and addictive behavior. The *rs6265* and *rs1799978* SNPs were the preferred choices for the purpose of this investigation because of their association with substance use and psychiatric disorders among various clinical populations [33–35, 50–52]. We isolated DNA from whole blood and performed genotyping using the Applied Biosystems^®^ ViiA™ 7 Real-Time PCR System (Life Technologies Corp., Carlsbad, CA, USA) with Applied Biosystems TaqMan Genotyping Master Mix (Life Technologies Corp.), as described previously [49]. The genotype call rates were 97.7 and 99.2 % for *BDNFrs6265* and *DRD2 rs1799978*, respectively.

### Urine toxicology

All participants underwent qualitative and semi-quantitative urine analysis weekly/biweekly using the iMDx™ Analyzer and Prep Assay (NOVX Systems Inc., Richmond Hill, ON, Canada). The urine toxicology assays were implemented as part of the treatment model to monitor methadone adherence and to identify use of opioids. The iMDx™ test can differentiate between natural and synthetic opioids, allowing for easier identification of specific opioid use. Urine samples were collected and assayed at the respective methadone clinic sites.

### Statistical analysis

Sample demographics were summarized using descriptive summary measures expressed as mean (standard deviation, SD) for continuous variables and number (percent) for categorical variables. Genotype and allele frequencies were computed and tested for Hardy–Weinberg equilibrium.

We performed univariate analysis on sample characteristics to evaluate differences between responders and nonresponders. Student’s *t* test was used for mean differences, and Chi square was used for categorical variables. We chose to use the results from these comparisons and include significant variables as covariates in our logistic regression model. We performed multivariable logistic regression analysis, with opioid use as the binary dependent variable and the two genetic variants, *BDNFrs6265* (A/A vs. A/G vs. G/G) and *DRD2 rs1799978* (G/A vs. A/A), as independent categorical variables adjusting for age, sex, ethnicity, methadone dose (mg), and duration of treatment (months). We also adjusted for the total number of urine screens to eliminate any effect of more frequent urine sampling, suggesting problematic behavior throughout treatment (change in outcome per opioid screen). We classified continued opioid use as having <80 % negative opioid urine screens (treatment nonresponders). This classification was based on data from our current sample and from previous literature demonstrating that 30–80 % of opioid urine screens generally test negative throughout the course of methadone treatment [53–55]. Given the maximum value of this range, individuals with greater than 80 % negative screens (or alternatively, less than 20 % positive screens) are considered to be in good standing and, therefore, responding well to treatment. Given that 85 % of the participants were of self-reported European origin, we did not perform subgroup analyses based on ethnicity due to the small sample size of other ethnic groups in our study; this ethnic distribution is in keeping with our region population mix. In our regression, participants of European origin were compared to non-European origin, and men were compared to women.

Regression results, including model coefficients (odds ratio, OR), corresponding CIs, and associated *p* values are reported. The criterion for statistical significance was set at alpha = 0.05. There were no missing data in our analyses. We performed all statistics using STATA Version 12 (StataCorp LP, College Station, USA). The study is reported in adherence with the Strengthening the Reporting of Observational Studies in Epidemiology statement [56].

We confirmed the statistical power of this investigation post hoc using Quanto Version 1.2.4 (Morrison & Gauderman 2009, California, USA), with treatment response (continued opioid use) as the outcome variable. Using an additive, gene-only, unmatched case–control (1:2) model, including 240 methadone patients and a two-sided test with a *p* = 0.05 level of significance, we had 85 % power to evaluate the effect of *BDNF rs6265* (minor allele frequency, MAF: 0.20) on methadone treatment response, with an OR of 1.5; and 70 % power to examine the effect of *DRD2 rs1799978* (MAF: 0.05) on methadone treatment response, with an OR of 1.8.

## Results

### Sample demographics

**Fig. 1:**
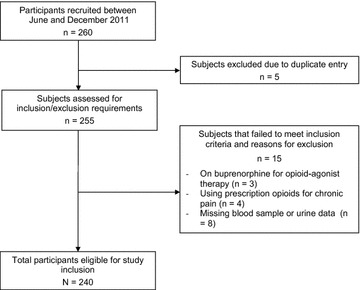
Flow diagram for participants included in study. Number of participants included at each stage of the study process and reasons for participant exclusion

Of the initial 260 participants recruited from methadone clinics, 20 participants were excluded from the study (duplicate entries = 5, buprenorphine treatment = 3, missing blood sample or urine data = 8, being prescribed opioids for chronic pain condition = 4). Therefore, 240 participants in total were included in the analysis (Fig. 1). The sample consisted of 144 (60.0 %) men and 96 (40.0 %) women, with a total mean age of 37.1 (SD = 10.4). Participants of European ethnicity made up 85 % of the sample. A majority of participants (81.3 %; n = 195) reported having a family history of mental illness or addiction. Responders and nonresponders were comparable across the majority of factors. Additional details of sample characteristics are shown in Table 1.

**Table 1:** Characteristics of patients on methadone treatment for opioid use disorder

Characteristic	Total (N = 240)	Responders (n = 167)	Nonresponders (n = 73)	*p* value
Age in years; mean (SD)	37.1 (10.4)	37.1 (10.7)	36.9 (9.6)	0.868
Male; n (%)	144 (60.0)	105 (62.9)	39 (53.4)	0.169
Married/common law; n (%)	93 (38.8)	66 (39.5)	27 (37.0)	0.711
Employed; n (%)	72 (30.0)	53 (31.7)	19 (26.0)	0.375
Completed post-secondary education; n (%)	81 (33.8)	52 (31.1)	29 (39.7)	0.196
Ethnicity				
European; n (%)	203 (84.6)	139 (83.2)	64 (87.7)	0.381
Native North/South American; n (%)	19 (7.9)	14 (8.4)	5 (6.8)	0.686
Asian; n (%)	2 (0.8)	2 (1.2)	0 (0)	0.348
Persian; n (%)	1 (0.4)	1 (0.6)	0 (0)	0.508
Age of initial opioid use in years; mean (SD)	23.1 (9.2)	22.3 (8.8)	25.0 (9.8)	0.037
Current cigarette smokers; n (%)	214 (89.2)	145 (86.8)	69 (94.5)	0.210
Number of cigarettes smoked/day; mean (SD)	18.0 (10.1)	18.6 (10.5)	16.6 (9.2)	0.158
Psychiatric comorbidity, self-reported; n (%)	116 (48.3)	81 (48.5)	35 (47.9)	0.937
Family psychiatric history; n (%)	195 (81.3)	133 (79.6)	62 (84.9)	0.334
Alcohol use disorder; n (%)	42 (17.5)	29 (17.4)	13 (17.8)	0.934
Methadone dose (mg); mean (SD)	89.5 (60.8)	97.5 (67.5)	71.2 (35.9)	0.002
Duration of MMT (months); mean (SD)	40.5 (42.6)	44.2 (44.1)	31.8 (37.8)	0.042
Total number of opioid urine screens; mean (SD)	65.7 (23.7)	64.9 (20.7)	67.5 (29.6)	0.278
Opioid use (% positive urine screens); mean (SD)	18.9 (24.1)	5.4 (5.6)	49.8 (21.4)	<0.001
*BDNF rs6265* genotype frequencies; n (%)				
G/G	153 (63.8)	110 (65.9)	43 (58.9)	0.302
A/G	81 (33.8)	52 (31.1)	29 (39.7)	0.196
A/A	6 (2.5)	5 (3.0)	1 (1.4)	0.458
*DRD2 rs1799978* genotype frequencies; n (%)				
A/A	213 (88.8)	150 (89.8)	63 (86.3)	0.427
A/G	27 (11.3)	17 (10.2)	10 (13.7)	0.427

Frequency for *DRD2 rs1799978* G/G genotype is 0 % in this sample; therefore, descriptive statistics are not available

*BDNF* brain-derived neurotrophic factor, *DRD2* dopamine receptor D2, *SD* standard deviation, *MMT* methadone maintenance treatment

### Genotypic profile

Genotype frequencies for *rs6265* and *rs1799978* are presented in Table 1. They did not deviate significantly from Hardy–Weinberg equilibrium (*p* = 0.21 for *rs6265*; *p* = 0.36 for *rs1799978*), and MAFs were consistent with previous literature (0.20 for *rs6265* and 0.05 for *rs1799978*). Among our sample of 240 methadone patients, 36.3 % (n = 87) had at least one *rs6265* risk allele, and 11.3 % (n = 27) had at least one *rs1799978* risk allele.

### Genetic effect on opioid use during treatment

The continued use of opioids during methadone treatment was an indication of treatment nonresponse and can be measured objectively across samples. This allowed us to examine whether there was a genetic component to outcomes of methadone treatment. On average, 18.9 % (SD 24.1) of total urine screens throughout the duration of methadone treatment were positive for opioids in the total sample. Similar patterns were observed among genotype frequencies of responders and nonresponders (Table 1). Our logistic regression analysis showed that the minor alleles of *BDNF rs6265* and *DRD2 rs1799978* were not associated with continued opioid use during methadone treatment, while adjusting for age, sex, ethnicity, age of initial opioid use, methadone dose, duration of treatment, and total number of opioid urine screens (*rs6265*: OR 1.37, 95 % CI 0.792, 2.371, *p* = 0.260; *rs1799978*: OR 1.28, 95 % CI 0.511, 3.182, *p* = 0.603) (Table 2).

**Table 2:** Summary of multivariable regression results

Continued opioid use	OR	95 % CI	*p* value
Age: year	1.00	0.966, 1.038	0.928
Sex: male	0.64	0.349, 1.178	0.152
Ethnicity: European	1.83	0.715, 4.697	0.207
Age of initial opioid use: year	1.02	0.986, 1.060	0.235
Methadone dose: milligram	0.99	0.983, 0.998	0.016
Duration on treatment: month	1.00	0.987, 1.005	0.364
Total number of opioid screens	1.01	0.997, 1.024	0.148
*BDNF rs6265*: allele (A)	1.37	0.792, 2.371	0.260
*DRD2 rs1799978*: allele (G)	1.28	0.511, 3.182	0.603

LR χ^2^(7) = 20.23, Prob > χ^2^ = 0.0165, Psuedo R^2^ = 0.0728, Log likelihood = −128.822

*BDNF* brain-derived neurotrophic factor, *DRD2* dopamine receptor D2, *OR* odds ratio, *CI* confidence interval

## Discussion

Genetic association studies in addiction research aim to characterize genetic differences and variation in the processes that underly addiction and response to treatment. Patients with opioid use disorder have significant interindividual variability in their clinical response to treatment, which may be attributed in part to genetic factors. Variation in addiction-related genes (such as *BDNF* and *DRD2)* due to polymorphisms in the genetic sequence may confer susceptibility to continued opioid use while on methadone treatment for opioid use disorder.

### Summary of findings

In this study, we explored the effect of *BDNF rs6265* and *DRD2 rs1799978* polymorphisms on an important methadone treatment outcome, continued opioid use, which represents an objective measurement of response to treatment. In our sample of 240 methadone patients of primarily European origin, we were unable to confirm a role for these specific SNPs in continued opioid use during treatment.

Our findings are in line with a study by de Cid and colleagues, the only other study to examine the influence of *BDNF rs6265* in methadone treatment response [39]. They performed a haplotype analysis of 30 SNPs in the *BDNF* coding region, including *rs6265*, in a sample of 91 Caucasian individuals receiving methadone treatment for opioid use disorder. Grouping their sample into responders and nonresponders, they were unable to establish an effect of *rs6265* on response to methadone treatment [39], but found that a haplotype block containing this specific SNP appeared more frequently in nonresponders compared with responders. However, the generalizability of these findings is limited by small sample size, large CIs, and short period of urinalysis testing (previous four urine screens, or approximately 1 month) [39]. With respect to other methadone outcomes, another study demonstrated no effect of *rs6265* on methadone dose in a sample of 227 former heroin-dependent individuals in methadone treatment [38].

The *rs1799978* SNP of *DRD2* has only been examined in association with opioid dependence or methadone dose in two single-SNP and haplotype analyses. In both studies (by Hung et al. [40]; Doehring et al. [43]), the minor allele of *rs1799978* was more common in opioid users. However, Hung et al. [40] demonstrated that carriers of the minor llele also required higher methadone doses in their sample of 321 methadone patients, which was not a consistent finding in the study by Doehring and colleagues for a sample of 85 German drug users [43], thus suggesting a potential ancestral influence of *rs1799978* on methadone dose.

Although methadone dose was a significant predictor of continued opioid use in our regression analysis, this was an expected finding given the available evidence on methadone dosing in treatment [16, 57]. We included this variable to ensure that any effect found between the SNPs and continued opioid use was not explained by the relationship between methadone dose and continued opioid use.

### Implications

Contrary to what the current literature suggests, although there may be a potential role for both *BDNF rs6265* and *DRD2 rs1799978* in susceptibility to opioid use disorder, the present study shows that these variants do not appear to exert large effects on continued illicit opioid use during treatment with methadone. Treatment response may, however, be influenced by the collective genetic risk conferred through multiple SNPs across several different genes (a polygenic effect). It is also possible that the continued illicit use of opioids during methadone treatment may be a result of other clinically relevant factors (i.e., medical or psychiatric comorbidity, social circumstances, life stressors, etc.).

### Future directions

Given that there is little conclusive evidence to support a genetic impact on methadone treatment, there is a need for well-designed, powerful, genome-wide association studies to identify specific SNPs that are relevant to methadone treatment response. Perhaps with this information, we will be able to simultaneously examine multiple candidate genes to understand the genetic composition of polygenic psychiatric disorders. Implementing the use of a gene score to assess an individual’s genetic load may prove to be a promising approach to predicting and identifying those patients who require closer monitoring or alternate treatment strategies to overcome their continued opioid use.

Future research should focus on investigating these questions in larger samples and in various populations to ensure validity. Furthermore, a thorough examination of other nongenetic determinants of continued opioid use may prove useful for identifying problematic areas that require modifications in treatment delivery. Additionally, the genetic effects of withdrawal symptoms and adverse methadone events may be a promising area of study.

### Strengths and limitations

To our knowledge, this is one of few studies to investigate the potential for an allelic effect of *BDNF rs6265* and *DRD2 rs1799978* on continued illicit opioid use among a large sample of methadone patients. These factors have not been thoroughly investigated in the context of methadone treatment response (as measured objectively through urine toxicology screens), which highlights a novel direction of research in the treatment of opioid use disorder with opioid-agonist treatments. Through this analysis, we aim to stimulate further research into potential polygenic influences on methadone treatment outcomes, as well as to confirm our findings in a larger sample of methadone patients. The uniformity of our cohort, attributed to consistency in delivery of treatment and standard of care across CATC clinics, ensures representativeness of the entire methadone patient population across Ontario, and likely throughout all of Canada. Our objective selection and definition of outcome measurements—specifically, continued opioid use—is also a noteable strength of this study.

Despite our negative findings, this study should be replicated in a larger sample using multiple genes in order to confirm a lack of association between *BDNF rs6265* or *DRD2 rs1799978* and methadone treatment response. Because the frequencies of the minor alleles were relatively low in our sample, a larger sample size may be required to estimate with confidence the effect of these variants on continued illicit opioid use.

In summary, the present study has demonstrated a lack of association between the two genetic variants (*BDNF rs6265* and *DRD2 rs1799978)* and MMT response, contrary to what previously had been believed about the role of these variants in psychiatric disorders and addictive behavior. Further research with larger samples is needed to re-evaluate this question, as well as to investigate multiple genes simultaneously to assess polygenic effects on susceptibility to poor treatment response. Nevertheless, this study elucidates the potential for other nongenetic determinants that may contribute to continued opioid use during methadone treatment; it also brings attention to further questions regarding the role of genetics in addiction research.

## Abbreviations

MMT: methadone maintenance treatment
BDNF: brain-derived neurotrophic factor
SNP: single nucleotide polymorphism
DRD2: dopamine receptor D2
GENOA: GENetics of Opioid Addiction
CATC: Canadian Addiction Treatment Centres
OATC: Ontario Addiction Treatment Centres
HIREB: Hamilton Integrated Research Ethics Board
SD: standard deviation
OR: odds ratio
CI: confidence interval
MAF: minor allele frequency
